# A network meta-analysis of arthroscopic rotator cuff repair

**DOI:** 10.1186/s12893-023-02078-4

**Published:** 2023-07-13

**Authors:** Tianshu You, Siyu Wu, Xiaolan Ou, Ying Liu, Xu Wang

**Affiliations:** 1 School of Electrical Engineering and Computer, Jilin Jianzhu University, Changchun, Jilin Province China; 2 Department of Hand Surgery, the Second Hospital of Jilin University, Changchun, Jilin Province China; 3 Department of Cardiology, Jilin Province Hospital, Changchun, Jilin Province China

**Keywords:** Rotator cuff tear, Single-row repair, Double-row repair, Suture bridge repair, Platelet-rich plasma therapy, Bone marrow stimulation

## Abstract

**Objective:**

Rotator cuff tear is a common shoulder injury that often leads to serious limitations in daily life. Herein, a network Meta-analysis using frequency theory was performed to evaluate the clinical outcomes of five rotator cuff repair techniques, including single-row repair, double-row repair, suture bridge repair, platelet-rich plasma therapy, and bone marrow stimulation, thus guiding clinical decision-making on rotator cuff repair.

**Methods:**

PubMed, EMbase, The Cochrane Library, and Web of Science were searched for randomized controlled trials and cohort studies comparing rotator cuff repair techniques published from inception to May 2022. Combined analysis and quality assessment were performed using software STATA15.1 and Review Manager5.3.

**Results:**

A total of 51 articles were finally included, including 27 randomized controlled trials and 24 cohort studies. Results from the network Meta-analysis showed that: (1) In terms of the American Shoulder and Elbow Surgeons score, platelet-rich plasma therapy, double-row repair, bone marrow stimulation, and single-row repair were significantly better than suture bridge repair. (2) In terms of Constant score, bone marrow stimulation was significantly better than double-row repair, single-row repair, and suture bridge repair. (3) In terms of visual analog scale score, platelet-rich plasma therapy was significantly better than double-row repair and suture bridge repair. (4) In terms of the Shoulder Rating Scale of the University of California at Los Angeles score, platelet-rich plasma therapy and double-row repair were relatively better but not significantly different from the other treatments. (5) In terms of the risk of re-tear, the re-tear rate of platelet-rich plasma therapy and double-row repair was significantly lower than that of single-row repair and suture bridge repair.

**Conclusion:**

Based on the results of network Meta-analysis and surface under the cumulative ranking, platelet-rich plasma therapy, bone marrow stimulation, and double-row repair have good overall rehabilitation effects. It is recommended to choose appropriate repair techniques as per the actual clinical situation.

## Introduction

The rotator cuff is an important organ that stabilizes the humeral head on the glenoid and plays an important role in maintaining shoulder flexion and abduction. Rotator cuff tear is a common rotator cuff injury, mostly in middle-aged and elderly patients, which is mainly manifested as shoulder pain and shoulder joint dysfunction [1, 2]. Long-term treatment will cause a huge economic burden to patients. Moreover, various complications caused by rotator cuff tears will seriously affect patients’ normal life. Rotator cuff injuries are a common form of sports injury. Conventionally, rehabilitation therapy is required for early mild injuries, while surgical treatment combined with rehabilitation is needed for severe injuries.

Arthroscopic rotator cuff repair has gradually replaced open repair as the major surgical procedure for rotator cuff repair due to its advantages of small incision [3]. Single-row repair has long been the standard approach used for rotator cuff tear repair, although some works have reported incomplete postoperative healing [4–6] or surgical failure [7] following single-row repair. To provide a better tendon-bone healing environment, double-row repair is preferred to increase the contact area of ​​the tendon and bone, increase the maximum load of the suture site, and reduce the risk of re-tear [8], which is widely favored by doctors and patients. A biomechanical study reported better initial fixation strength with double-row fixation compared to single-row fixation [9]. However, there are also a series of randomized controlled studies that indicate no statistical difference in clinical outcomes between the two methods. The single-row repair is simple and time-saving and can eliminate certain potential risks, especially for patients with small tears. However, for medium and large injuries, the single-row repair often has no ideal prognostic outcomes. In another randomized controlled study, MRI results showed that double-row repair was significantly better than single-row repair for rotator cuff tears with a size of > 30 mm [10].

Suture bridge repair combines the merits and demerits of single-row repair and double-row repair [11]. On the basis of double-row repair, the outer-row mesh structure is used to fix the footprint area, and the rotator cuff tissue is squeezed and fixed through the thread bridge, to obtain a larger area of ​​contact between the tendon and bone, thereby accelerating healing. Although existing studies have shown that suture bridge repair can achieve better biomechanical properties than double-row repair, there is no sufficient clinical evidence to confirm that it has better postoperative efficacy. Therefore, further exploration is needed.

For a better surgical prognosis in rotator cuff repair and reducing the risk of postoperative re-tear, the use of adjuvant biologics has been considered. Platelet-rich plasma (PRP), a biological preparation developed in recent years, is obtained by centrifugation of whole blood, containing a variety of growth factors, which can accelerate the formation of new blood vessels and the proliferation of stem cells, promote the formation of bone matrix, improve the metabolic rate, promote tendon repair, and effectively prevent the occurrence of re-tear [12]. PRP has been widely used in medical operations. Some scholars have proposed that the efficacy of PRP depends to a certain extent on the source, preparation method, dosage, and administration regimen [13]. Given this, a more complete preparation system can make it better exert its medicinal value. Bone marrow stimulation technologies such as multi-channel technology and microfracture technology are a group of new tendon repair technologies, which are simple and easy to operate, reduce the risk of in vitro infection caused by injection of growth factors such as PRP, and quickly provide sufficient mesenchymal stem cells for tendon healing. Compared with traditional arthroscopic repair, bone marrow stimulation has better biomechanical properties. A Meta-analysis [14] has shown that bone marrow stimulation can effectively reduce the risk of re-tear, but postoperatively, the rotator cuff function has not been significantly improved. In addition, there are few published studies on bone marrow stimulation, and its repair ability remains to be further verified.

In this study, the network Meta-analysis method was used to compare the efficacy of single-row repair, double-row repair, suture bridge repair, PRP therapy, and bone marrow stimulation for the treatment of rotator cuff tears. We attempted to provide evidence-based medicine support for the optimal therapeutic regimen for the clinical repair of rotator cuff tears.

## Data and methods

### Search strategy

Using “rotator cuff injuries, single-row, double-row, suture-bridge, platelet-rich plasma and bone marrow stimulation” as keywords, literature retrieval was performed in PubMed, Embase, The Cochrane Library, and Web of Science. The retrieval time was from inception to May 1, 2022.

### Inclusion and exclusion criteria

Inclusion criteria for literature retrieval were (1) Type of study: randomized controlled trial or cohort study; (2) Subjects: patients diagnosed with rotator cuff tear and followed up for ≥ 6 months; (3) Interventions: at least any two of single-row repair, double-row repair, suture bridge repair, PRP therapy, and bone marrow stimulation; (4) Outcome indicators: at least any one of re-tear rate, Constant shoulder score (Constant) score [15], The Shoulder Rating Scale of the University of California at Los Angeles (UCLA) [16], the American Shoulder and Elbow Surgeons (ASES) score [17], and Visual Analog Scale (VAS).

Exclusion criteria included (1) retrospective studies, literature reviews, or conference papers with full text not available; (2) trials with no use of arthroscopy or suture anchoring techniques; (3) trials that did not involve imaging for structural assessment; and (4) animal or cell experiments.

### Data extraction

Relevant literature was searched as per the PRISMA statement. To ensure the accuracy of the data and the rigor of the research, two researchers independently extracted relevant data and then cross-checked them according to the previously formulated standards. If disagreements occurred, the decision regarding data extraction was done by the third reviewer. The extracted baseline data included: the lead author’s name, publication time, number of patients, patient’s sex, mean age, interventions, follow-up time, and tear size.

### Literature quality assessment

The Cochrane’s Risk of Bias tool was used to assess the risk of bias in the included randomized controlled trials, involving six domains: selection bias (random allocation sequence generation, allocation concealment), performance bias, detection bias, attrition bias, reporting bias, and other forms of bias. Each domain for assessing the risk of bias was assessed as low risk, high risk, or unclear risk, and then analyzed using Review Manager 5.3. The Newcastle–Ottawa Scale, which contains eight items, with a maximum score of 9, was used to evaluate the quality of the included cohort studies. A higher score indicates a lower risk of bias. If there was any disagreement, the third reviewer was consulted.

### Outcome indicators

The outcome indicators included: AESE score, Constant score, VAS score, UCLA score, and re-tear rate.

Constant score is an important score for orthopedic surgeons to evaluate patients' shoulder function. It contains subjective and objective scores, involving eight aspects. The full score is 100 points. Higher scores indicate better recovery of shoulder function.

UCLA score acts as the final censoring score for shoulder repair, in which indicators such as pain and satisfaction are subjectively scored by patients, and indicators such as active forward elevation and strength are scored objectively by doctors, with a full score of 35 points. Higher scores indicate better outcomes and higher patient satisfaction.

AESE score is a patient-determined assessment scale for shoulder function and contains two dimensions of shoulder function: pain and performance in activities of daily living. It is a 100-point scale. Higher scores indicate better shoulder function.

VAS score is a commonly used quantitative index to measure the intensity of pain, usually providing a range of scores from 0–10. Lower scores indicate less pain intensity.

### Statistical analysis

STATA15.1 and Review Manager5.3 were used for combined analysis and quality assessment, and a frequentist random-effects model was used for network Meta-analysis. The odds ratio (OR) was used as the effect size for dichotomous data (re-tear rate); mean difference (MD) was used as the effect size for continuous data such as the Constant score, UCLA score, and ASES score. The point estimates for each effect size and its 95% confidence interval (95% CI) were obtained. An inconsistency test was carried out by node splitting method on the outcome indicators containing closed loops in the evidence network graph. If a value of *P* > 0.05, the inconsistency was insignificant. The results of direct and indirect comparisons could be combined for analysis. On the contrary, the source of inconsistency was searched and eliminated, and the data were then merged and analyzed. As per the surface under the cumulative ranking (SUCRA) curve, the ranking results of the intervention measures were obtained. The larger area under the curve indicates better ranking and more effective intervention measures. Funnel plots were finally utilized to check for the existence of publication biases.

## Results

### Literature retrieval results

**Fig. 1:**
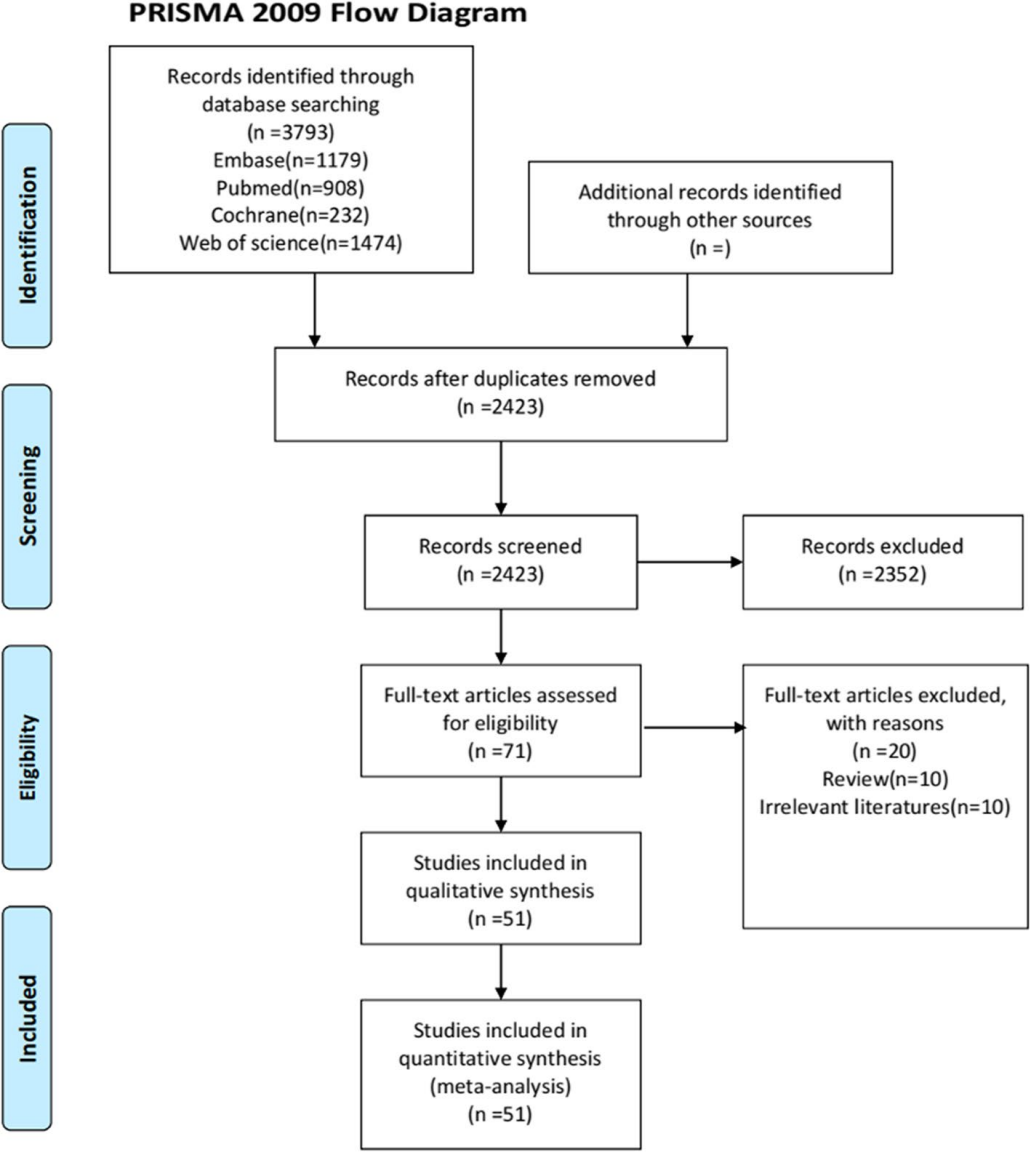
Flow chart of document screening

As shown in Fig. 1, a total of 3,793 related articles (908 in PubMed, 1,179 in Embase, 1,474 in Web of Science, and 232 in Cochrane Library) were initially retrieved. There were 2,423 articles after excluding duplicate articles. The 71 of 2,423 articles were screened out by reading the titles and abstracts. Of these 71 articles, 20 articles that did not meet the inclusion criteria were excluded after reading the full text and the remaining 51 articles were finally included for review [18–68]. There were 27 randomized controlled trials and 24 cohort studies included (Table 1).

**Table 1:** Characteristics of selected trials

id	Study	Comparison	No. of patients	Age(years)	No. of male	Follow-up(month)
1	Burks2009	SR/DR	20/20	56/57	——	22.5
2	Grasso2009	SR/DR	37/35	58.3 /55.2	16/18	24.8
3	Aydin2010	SR/DR	34/34	59/57	——	36
4	Koh2011	SR/DR	31/31	61.6/61.1	9/11	31/32.8
5	Lapner2012	SR/DR	48/42	56/57.8	13/13	24
6	Carbonel2012	SR/DR	80/80	55.79/55.21	35/33	24
7	Ma2012	SR/DR	27/26	60.8/61.6	15/14	33.3/33.5
8	Nicholas2016	SR/DR	20/16	61/65	11/12	26
9	Francesch2016i	SR/DR	30/28	61.8/58.9	12/15	——
10	Wade2017	SR/DR	28/28	55.39/53.18	18/21	——
11	Imam2020	SR/DR	40/40	61.6/60.0	——	36
12	Zafra2020	SR/SB	25/25	50.9/54.1	11/12	32.5
13	Pandey2016	SR/PRP	50/52	54.1/54.8	36/38	24
14	Jo2015	SB/PRP	37/37	60.92/60.08	9/8	24
15	Randelli2011	SR/PRP	23/22	59.5/61.6	13/8	24
16	Malavolta2014	SR/PRP	27/27	54.07/55.30	9/8	24
17	Holtby2016	SR/PRP	41/41	59/59	21/20	6
18	D ‘Ambrosi2016	SR/PRP	20/20	62/57.9	10/9	6
19	Jo2013	SB/PRP	24/24	61.92/64.21	14/10	17.26/15.88
20	Osti2013	SR/BMS	29/28	59.8/61.2	13/16	29/29
21	Milano2013	SR/BMS	38/35	63.1/60.6	19/22	——
22	Ebert2017	DR/PRP	28/27	59.7/59.5	17/11	42.7/41.5
23	Malavolta2018	SR/PRP	25/26	54.0/55.4	9/8	63. 1/60.6
24	Wang2015	DR/PRP	30/30	58.4/59.8	17/11	4
25	Flury2016	DR/PRP	60/60	58.9/57.8	18/20	24
26	Turan2021	SR/DR	20/23	54.95/60.83	4/5	29.9/27.57
27	Pulatkan2019	SR/DR/BMS	40/39/44	59.2/60.6/58.1	15/11/11	30/29/30
28	Plachel2020	SR/DR	16/11	60/62	12/7	13/12
29	Chen2019	SR/DR	52/53	57.5/56.7	18/14	31/29
30	Hantes2017	SR/DR	34/32	49.4/51.2	22/23	——
31	Zhou2017	SR/DR/SB	115/163/75	57.5/56.9/55.9	——	30
32	Wang2015	SR/DR	146/102	57.2/58.4	56/39	——
33	Tudisco2013	SR/DR	20/20	66/63	13/12	40/39
34	Denard2012	SR/DR	45/62	61.2/62.0	33/35	110.7/82.8
35	Park2008	SR/DR	40/38	57/54.4	20/22	——
36	Sugaya2005	SR/DR	39/41	57.7/58.1	28/28	41.3
37	Li2021	SR/PRP	9/10	55.7/57.35	22/20	24.7/24.87
38	Martinelli2019	SR/PRP	11/11	51.4/57	8/7	12/12
39	Zhang2016	DR/PRP	30/30	57.2/56.9	16/15	12.1/11.7
40	Gwinner2016	DR/PRP	18/18	61.2/61.2	11/8	24.4/24.6
41	Panella2016	SR/SB	24/20	58.0/58.7	——	24/24
42	Park2014	SR/SB	118/103	——	——	6/6
43	Kakoi2018	DR/SB	35/39	66.1/62.9	26/28	17.1/15.5
44	Hashiguch2018i	DR/SB	52/63	61.6/62.1	28/37	37.2/35.14
45	Kim2011	DR/SB	26/26	57.46/58.96	16/14	37.2
46	Kim2020	SR/BMS	42/56	64.2/64.6	23/26	24
47	Yoon2016	SB/BMS	54/21	62.8/64.95	26/9	24.37/25.47
48	Liu2021	DR/PRP	48/24	63.3/63.9	24/10	50.5/50.1
49	Jo2013	DR/BMS	67/57	60.1/58.89	33/25	41.04/31.79
50	Li,c2021	SR/DR	34/34	56.7/58.2	14/16	39/41
51	Sánchez2021	SR/PRP	18/17	52.3/53.7	14/9	12

### Methodological quality of the included studies

**Fig. 2:**
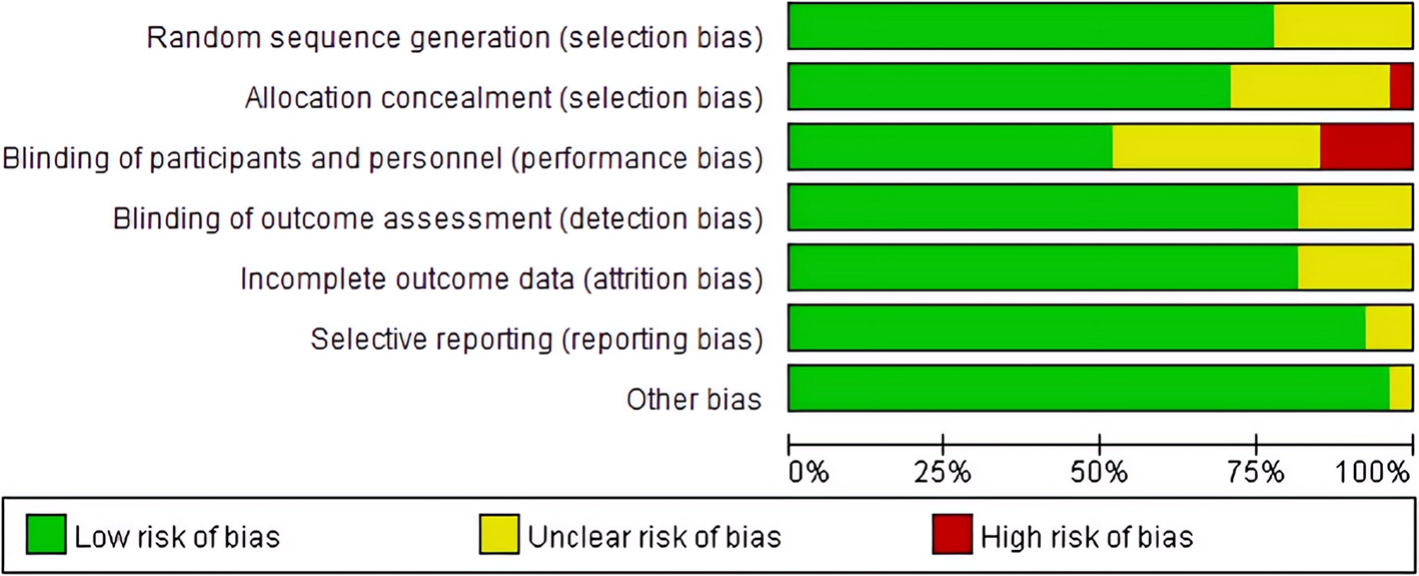
Risk of bias for randomized controlled trials

Quality evaluation of the included studies indicated that of the 27 randomized controlled trials, 16 studies produced correct randomization sequences; 16 studies correctly concealed randomization sequences; 11 studies had no performance bias and 3 studies had performance bias; 19 studies had blinded assessments, with no detection bias; 17 studies reported complete outcome data, with no attrition bias; 20 studies made a statement about their findings; 21 studies had no other forms of bias (Fig. 2).

The quality of the included cohort studies was assessed using the Newcastle–Ottawa Scale. There are eight items in total, with a full score of 9 points. Higher scores indicate lower risks of bias. There were 19 studies with the total score of over 7 points. Therefore, the overall reliability of the included cohort studies was high.

### Network met-analysis results

#### ASES score

**Fig. 3:**
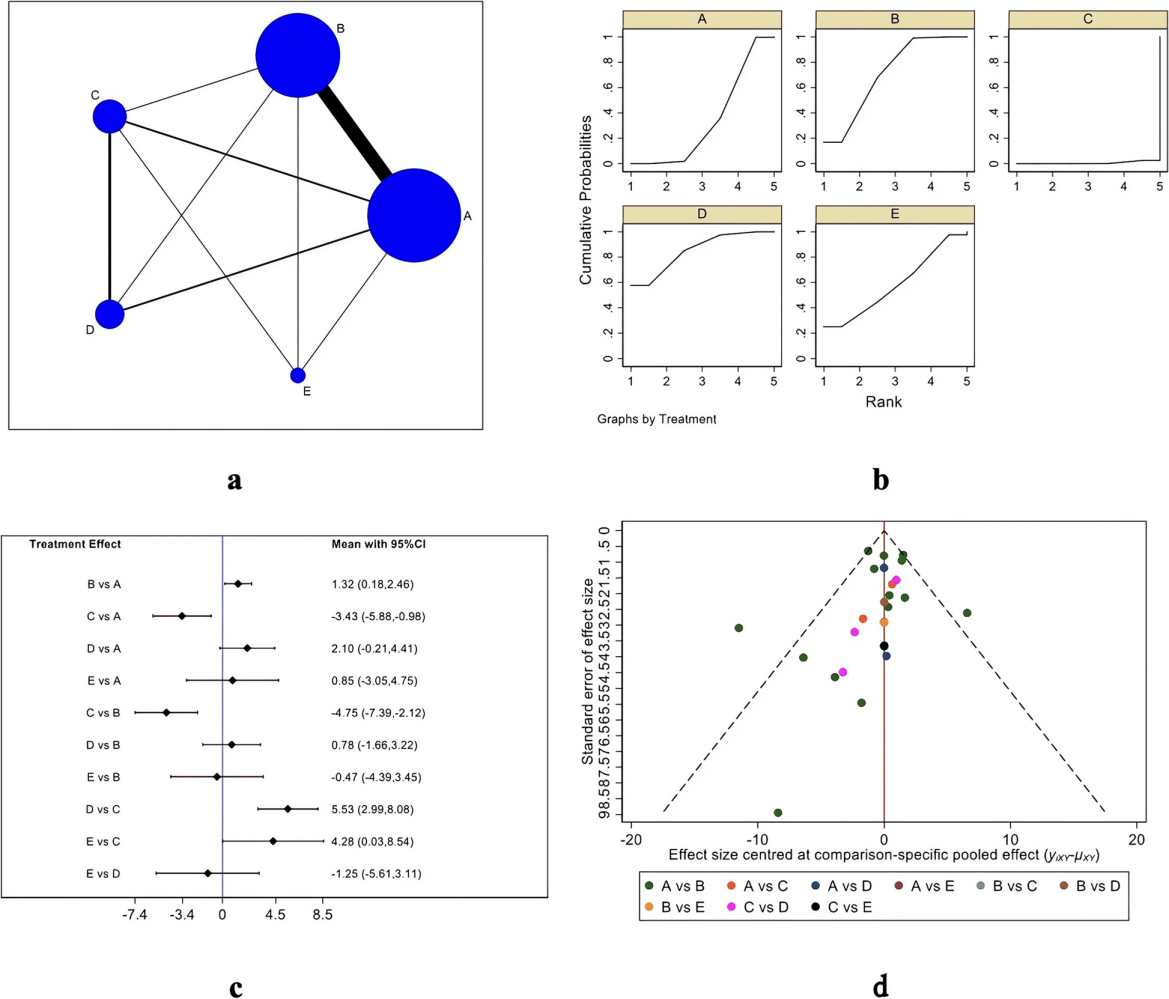
ASES scoring results (**a** Network Diagram; **b** Cumulative probability plot; **c** Forest map; **d** funnel chart)

Figure 3 shows the ASES score evaluation system. Figure 3A indicates the evidence network diagram of the ASES score. The outcome index involved five treatment measures, namely single-row, double-row, suture bridge, PRP therapy, and bone marrow stimulation, including 26 studies in total [18, 21–25, 27, 29–31, 34, 36, 42, 45, 46, 49, 51–53, 59, 62–66]. The included studies were first tested for inconsistency (*P* = 0.6350 > 0.05) and then for local inconsistency tests using the node-splitting method. Both the testing results were insignificant and therefore, the consistency model could be used for analysis. Figure 3B shows the SUCRA chart of ASES scores for five interventional measures. When both randomized controlled trials and cohort studies were included, the ASES scores of the five interventions were ranked as follows: PRP (SUCRA = 85.2), double-row (SUCRA = 71.0), bone marrow stimulation (SUCRA = 58.7), single-row (SUCRA = 34.4), and suture bridge (SUCRA = 0.7). Table 2 and Fig. 3C describe the trapezoidal comparison table of every two interventions and its intuitive forest diagram, respectively. Assessment of ASES scores indicated the efficacy of PRP therapy, double-row repair, bone marrow stimulation, and single-row repair was significantly better than that of suture bridge repair, and double-row repair, as well as the efficacy of double-row repair was significantly better than that of single-row repair, but there was no significant statistical difference among PRP therapy, double-row repair and bone marrow stimulation. A funnel plot was made for the obtained results to evaluate the possible publication bias of the ASES score (Fig. 1D), which is basically symmetrical, indicating no significant publication bias.

**Table 2:** ASES Score ladder table

PRP	-0.78 (-3.22,1.66)	-1.25 (-5.61,3.11)	-2.10 (-4.41,0.21)	-5.53 (-8.08,-2.99)
0.78 (-1.66,3.22)	DR	-0.47 (-4.39,3.45)	-1.32 (-2.46,-0.18)	-4.75 (-7.39,-2.12)
1.25 (-3.11,5.61)	0.47 (-3.45,4.39)	BMS	-0.85 (-4.75,3.05)	-4.28 (-8.54,-0.03)
2.10 (-0.21,4.41)	1.32 (0.18,2.46)	0.85 (-3.05,4.75)	SR	-3.43 (-5.88,-0.98)
5.53 (2.99,8.08)	4.75 (2.12,7.39)	4.28 (0.03,8.54)	3.43 (0.98,5.88)	SB

#### Constant score

**Fig. 4:**
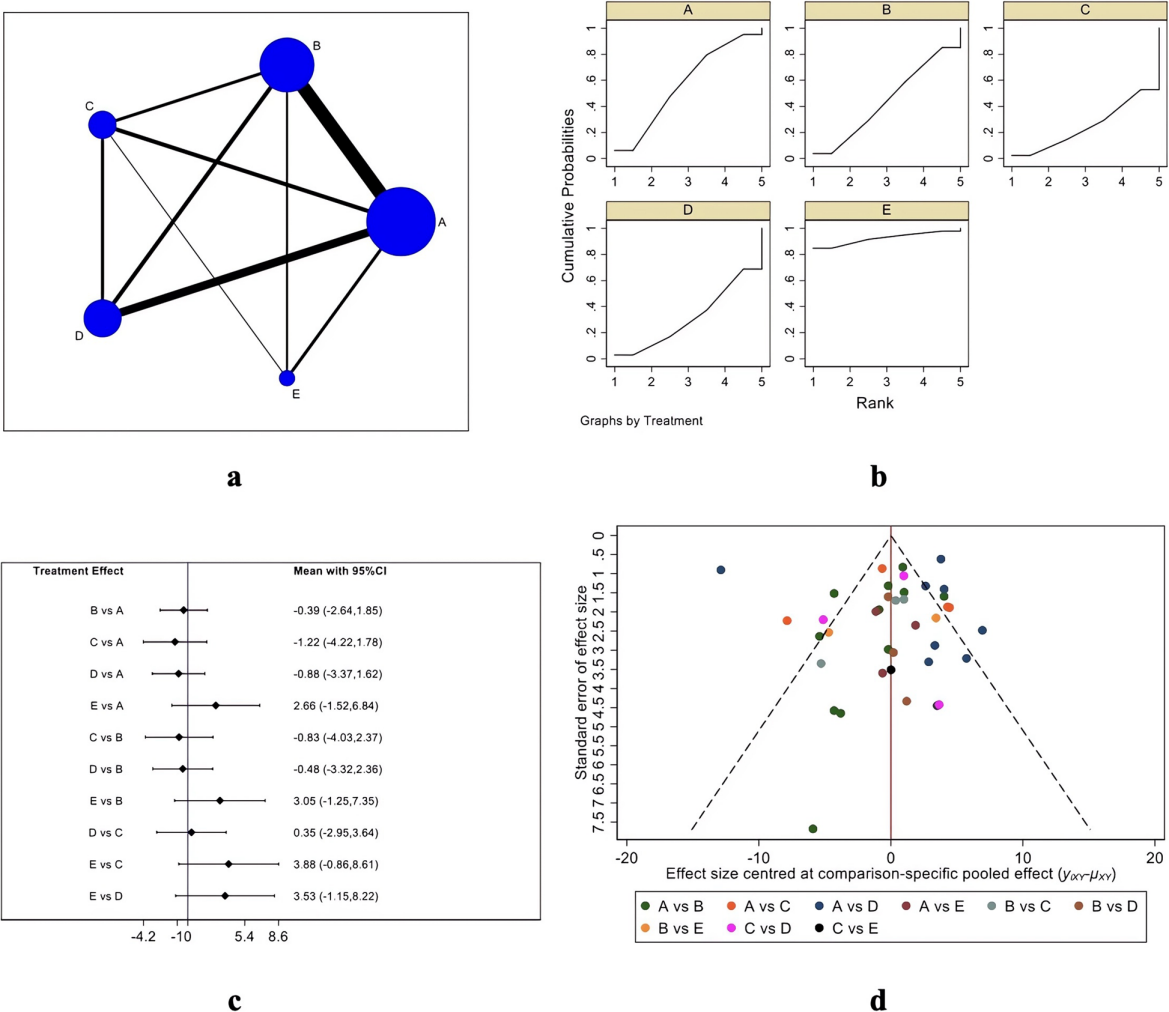
Constant scoring results (**a** Network Diagram; **b** Cumulative probability plot; **c** Forest map; **d** funnel chart)

Figure 4 depicts the Constant score evaluation system, in which Fig. 4A is the evidence network diagram of the Constant score. This outcome index involved five treatment measures, namely single-row, double-row, suture bridge, PRP, and bone marrow stimulation, including a total of 36 studies [18–33, 35–38, 40, 42, 44–46, 48–52, 54–59, 61–63, 65, 67]. The obtained studies were first tested for inconsistency (*P* = 0.8267 > 0.05) and then for local inconsistency tests. Both of the testing results were insignificant and therefore, the consistency model was used for analysis. Figure 4B is the SUCRA chart of Constant scores for the five interventional measures. When both randomized controlled trials and cohort studies were included, the Constant scores of the five interventions were ordered: bone marrow stimulation (SUCRA = 92.4), single-row (SUCRA = 57.1), double-row (SUCRA = 44.3), PRP (SUCRA = 31.4), and suture bridge (SUCRA = 24.8) Fig. 4C is the forest plot for the comparison between every two measures, and Table 3 is the trapezoidal tale for the comparison between every two measures. There were no significant differences in the Constant score between the five treatment measures. As shown in Fig. 4D, the funnel plot is basically symmetrical, indicating no significant publication bias.

**Table 3:** Constant Score ladder table

BMS	-2.66 (-6.84,1.52)	-3.05 (-7.35,1.25)	-3.53 (-8.22,1.15)	-3.88 (-8.61,0.86)
2.66 (-1.52,6.84)	SR	-0.39 (-2.64,1.85)	-0.88 (-3.37,1.62)	-1.22 (-4.22,1.78)
3.05 (-1.25,7.35)	0.39 (-1.85,2.64)	DR	-0.48 (-3.32,2.36)	-0.83 (-4.03,2.37)
3.53 (-1.15,8.22)	0.88 (-1.62,3.37)	0.48 (-2.36,3.32)	PRP	-0.35 (-3.64,2.95)
3.88 (-0.86,8.61)	1.22 (-1.78,4.22)	0.83 (-2.37,4.03)	0.35 (-2.95,3.64)	SB

#### VAS score

**Fig. 5:**
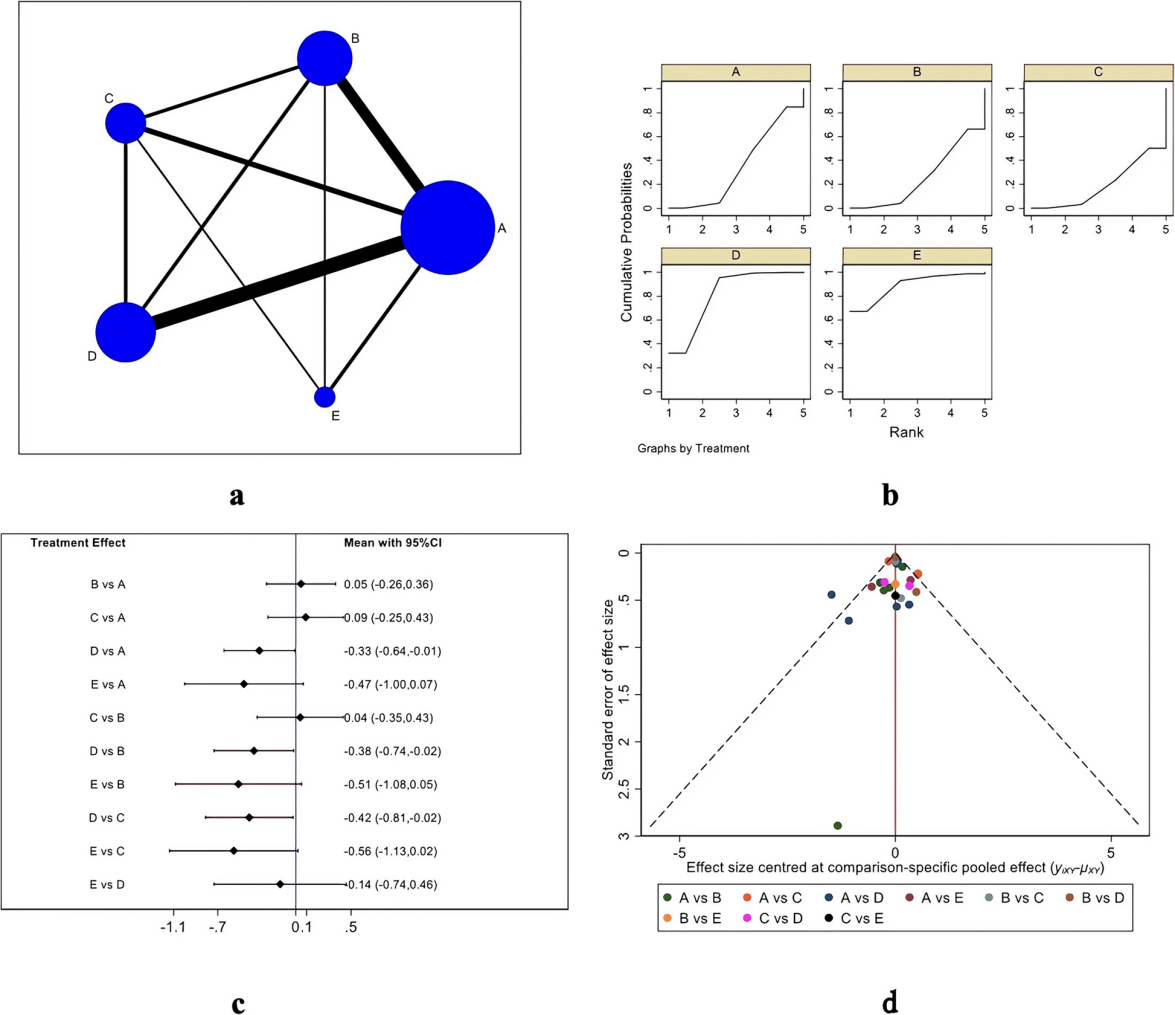
VAS scoring results (**a** Network Diagram; **b** Cumulative probability plot; **c** Forest map; **d** funnel chart)

Figure 5 indicates the VAS score evaluation system, in which Fig. 5A is the evidence network diagram of the VAS score. This outcome index involved five treatment measures, namely single-row, double-row, suture bridge, PRP, and bone marrow stimulation, and a total of 21 studies were included [28–33, 35, 40, 41, 44, 48, 49, 54–56, 59, 61–64, 66]. The data extracted from the 21 studies were tested for inconsistency (*P* = 0.0000 < 0.05) and then for local inconsistency using the node-splitting method to seek the source of inconsistency. We found that the inconsistency originated from two studies, Li [54] and Martinelli [55]. After excluding these two studies, the value of *P* = 0.2125 > 0.05 indicated that consistency existed and then the consistency model could be used. Figure 5B is the SUCRA chart of VAS scores for the five interventional measures. When both randomized controlled trials and cohort studies were included, the VAS scores of the five interventions were ranked: bone marrow stimulation (SUCRA = 88.9), PRP (SUCRA = 81.7), single-row (SUCRA = 34.5), double-row (SUCRA = 25.5), and suture bridge (SUCRA = 19.3). Figure 5C and Table 4 are the forest plot and trapezoidal table for the comparison between every two treatment measures, respectively. In terms of VAS scores, the curative effect of PRP therapy was significantly better than that of single-row repair, double-row repair, and suture bridge repair (P < 0.05), and there was no significant difference between single-row repair, double-row repair, and suture bridge repair. A funnel plot was made for the obtained data to evaluate the possible publication bias of the VAS score. As shown in Fig. 5D, the funnel plot is basically symmetrical, indicating no obvious publication bias.

**Table 4:** VAS Score ladder table

BMS	0.14 (-0.46,0.74)	0.47 (-0.07,1.00)	0.51 (-0.05,1.08)	0.56 (-0.02,1.13)
-0.14 (-0.74,0.46)	PRP	0.33 (0.01,0.64)	0.38 (0.02,0.74)	0.42 (0.02,0.81)
-0.47 (-1.00,0.07)	-0.33 (-0.64,-0.01)	SR	0.05 (-0.26,0.36)	0.09 (-0.25,0.43)
-0.51 (-1.08,0.05)	-0.38 (-0.74,-0.02)	-0.05 (-0.36,0.26)	DR	0.04 (-0.35,0.43)
-0.56 (-1.13,0.02)	-0.42 (-0.81,-0.02)	-0.09 (-0.43,0.25)	-0.04 (-0.43,0.35)	SB

#### UCLA score

**Fig. 6:**
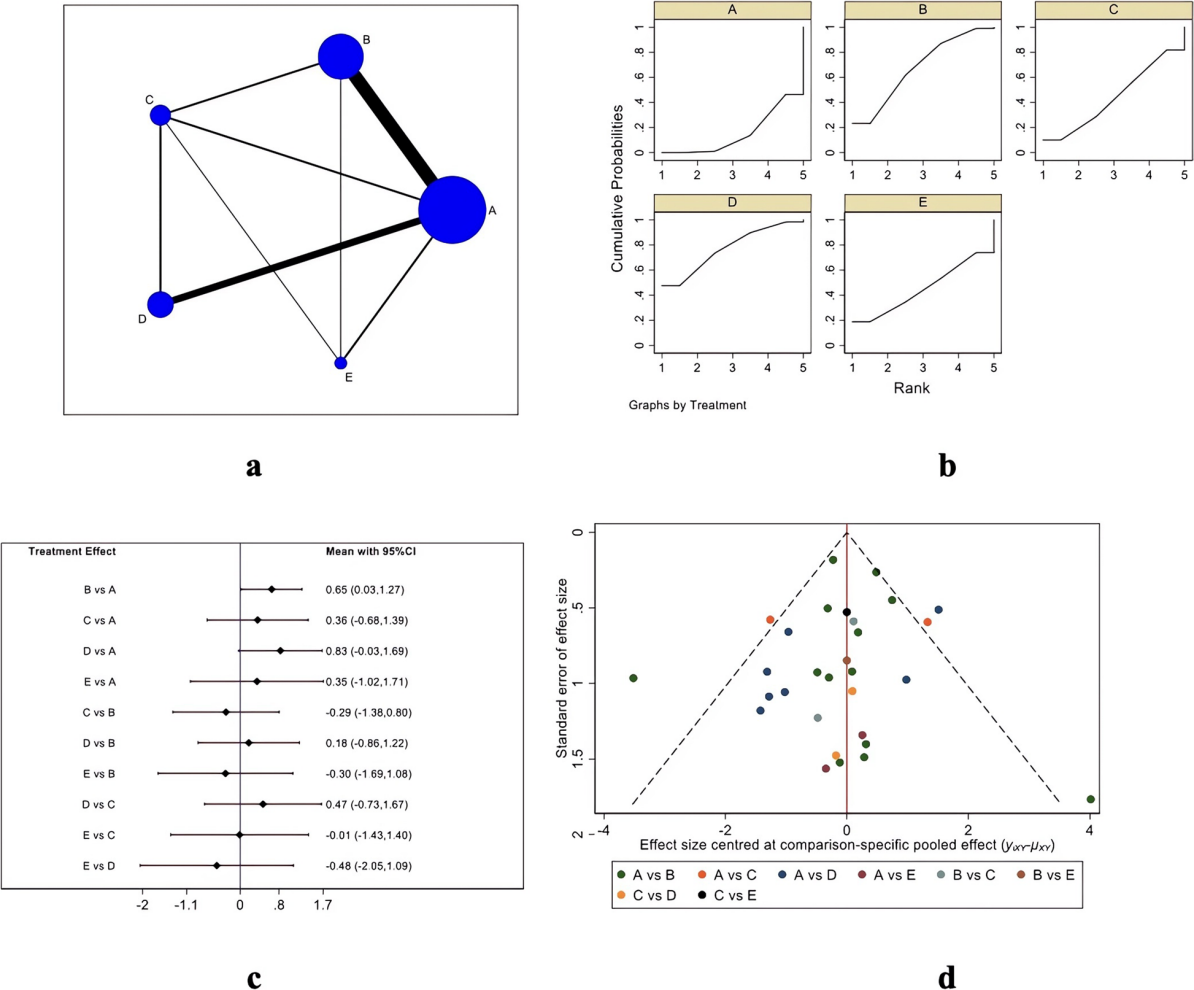
UCLA scoring results (**a** Network Diagram; **b** Cumulative probability plot; **c** Forest map; **d** funnel chart)

Figure 6 shows the UCLA score evaluation system, in which Fig. 6D is the evidence network diagram of the UCLA score. This outcome index involved five treatment measures, namely single-row, double-row, suture bridge, PRP, and bone marrow stimulation, and a total of 25 studies were included [18, 21, 23, 24, 27, 28, 30–33, 36, 37, 40, 43, 48, 49, 51, 53–55, 58, 61–63, 65, 66]. The data extracted from the 25 studies were first tested for inconsistency (*P* = 0.4922 > 0.05) and then for local inconsistency using the node-splitting method. Results from both inconsistency tests were insignificant, indicating that the consistency model could be used for analysis. Figure 6B is the SUCRA chart of UCLA scores for the five interventional measures. When both randomized controlled trials and cohort studies were included, the UCLA score results of the five interventions were ranked: PRP (SUCRA = 77.4), double-row (SUCRA = 67.9), bone marrow stimulation (SUCRA = 45.3), suture bridge (SUCRA = 44.2), and single-row (SUCRA = 15.3). Figure 6C and Table 5 are the forest plot and trapezoid table for the comparison between every two measures. The curative effect of double-row repair was significantly better than that of single-row repair. Although the postoperative UCLA score of PRP therapy ranked in the forefront, there was no significant difference in the curative efficacy between PRP and the other measures. A funnel plot was made for the obtained data to evaluate the possible publication bias of the UCLA score. As shown in Fig. 6D, the funnel plot is basically symmetrical, indicating no significant publication bias.

**Table 5:** UCLA Score ladder table

PRP	-0.18 (-1.22,0.86)	-0.48 (-2.05,1.09)	-0.47 (-1.67,0.73)	-0.83 (-1.69,0.03)
0.18 (-0.86,1.22)	DR	-0.30 (-1.69,1.08)	-0.29 (-1.38,0.80)	-0.65 (-1.27,-0.03)
0.48 (-1.09,2.05)	0.30 (-1.08,1.69)	BMS	0.01 (-1.40,1.43)	-0.35 (-1.71,1.02)
0.47 (-0.73,1.67)	0.29 (-0.80,1.38)	-0.01 (-1.43,1.40)	SB	-0.36 (-1.39,0.68)
0.83 (-0.03,1.69)	0.65 (0.03,1.27)	0.35 (-1.02,1.71)	0.36 (-0.68,1.39)	SR

#### Re-tear risk

**Fig. 7:**
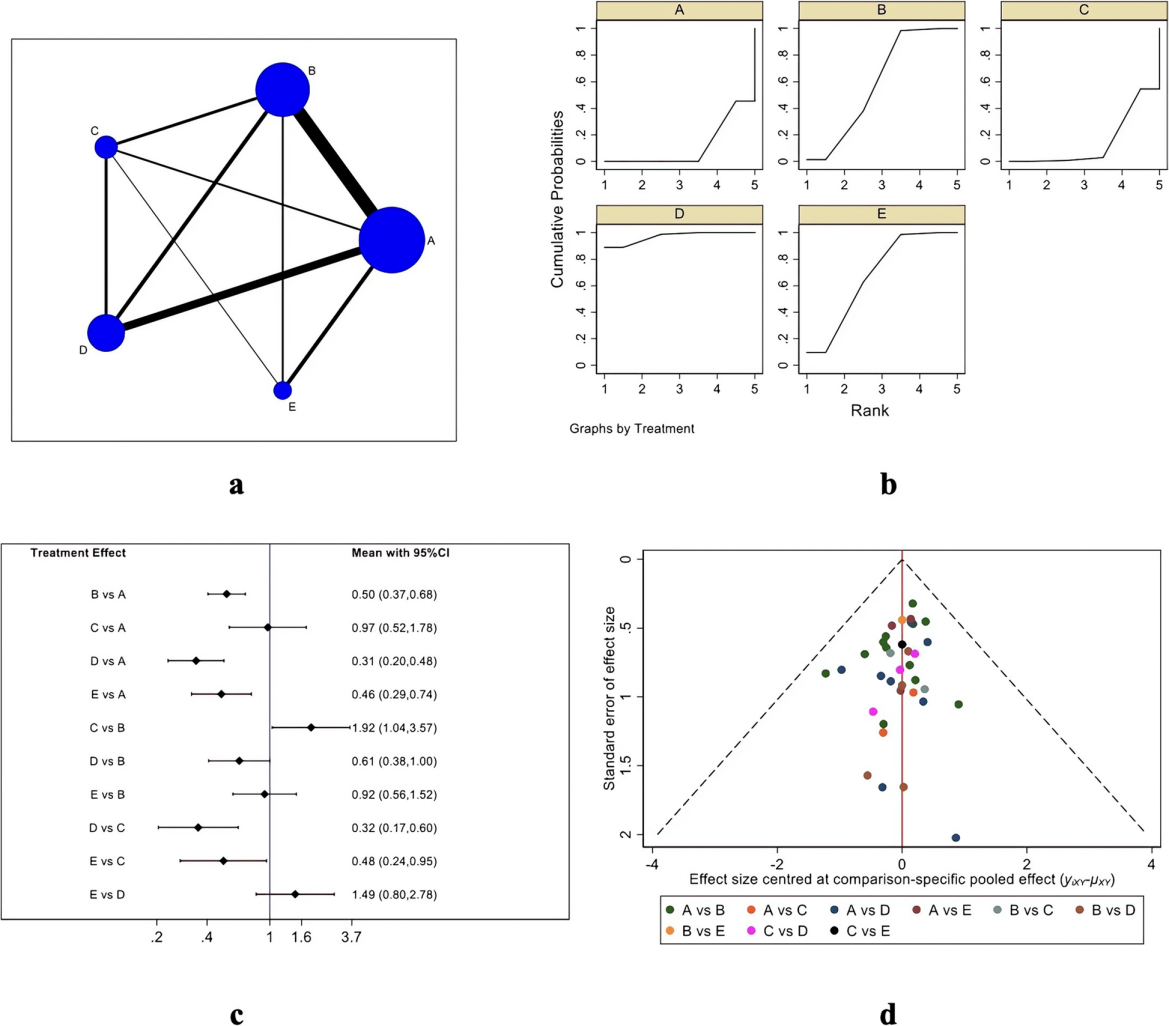
Re-tear risk results (**a** Network Diagram; **b** Cumulative probability plot; **c** Forest map; **d** funnel chart)

Figure 7 describes the re-tear risk evaluation system, in which Fig. 7A is the evidence network diagram of re-tear risk. This outcome index involved five treatment measures, namely single-row, double-row, suture bridge, PRP, and bone marrow stimulation, and a total of 38 studies were included [18, 21, 23, 24, 26, 27, 29–41, 44–47, 49, 50, 53–58, 60–65]. Data extracted from these studies were used for inconsistency tests (*P* = 0.0097 < 0.05). As inconsistency existed, local inconsistency tests were performed using the node-splitting method, to find the source of inconsistency. After elimination of Pulatkan [54] and Hashiguchi [61], the inconsistency test indicated no significance (*P* = 0.0519 > 0.05), and then the consistency model could be used for analysis. Figure 7B is the SUCRA chart of re-tear rates for the five interventional measures. When both randomized controlled trials and cohort studies were included, the re-tear rates for the five interventions were ranked: PRP (SUCRA = 96.8), bone marrow stimulation (SUCRA = 67.8), double-row (SUCRA = 59.5), -suture bridge (SUCRA = 14.2), and single-row (SUCRA = 11.7). Figure 7C and Table 6 are the forest plot and trapezoidal table for the comparison between every two treatment measures. The re-tear rate of PRP therapy, bone marrow stimulation, and double-row repair was significantly lower than that of single-row repair and suture bridge repair (P < 0.05), and there was no significant difference between PRP therapy, bone marrow stimulation, and double-row repair. A funnel plot was made for the obtained data to evaluate the possible publication bias of the re-tear risk. As shown in Fig. 7D, the funnel plot is basically symmetrical, indicating no obvious publication bias.

**Table 6:** Risk of re-tear ladder table

PRP	1.49 (0.80,2.78)	1.63 (1.00,2.64)	3.13 (1.66,5.91)	3.24 (2.08,5.05)
0.67 (0.36,1.24)	BMS	1.09 (0.66,1.80)	2.10 (1.05,4.16)	2.17 (1.35,3.48)
0.61 (0.38,1.00)	0.92 (0.56,1.52)	DR	1.92 (1.04,3.57)	1.99 (1.48,2.68)
0.32 (0.17,0.60)	0.48 (0.24,0.95)	0.52 (0.28,0.96)	SB	1.03 (0.56,1.91)
0.31 (0.20,0.48)	0.46 (0.29,0.74)	0.50 (0.37,0.68)	0.97 (0.52,1.78)	SR

## Discussion

Rotator cuff injury is a common shoulder disease. Traditional open surgery can result in many postoperative complications and slow recovery. At present, arthroscopic repair has gradually become the mainstream trend. However, which treatment measure is the best under arthroscopy? There is no conclusive conclusion yet. Herein, a network Meta-analysis was carried out to focus on the prognostic efficacy of five rotator cuff repair techniques based on the data extracted from relevant randomized controlled trials and cohort studies. Five rotator cuff repair techniques, including single-row repair, double-row repair, suture bridge repair, PRP therapy, and bone marrow stimulation, were ranked based on their prognostic outcomes. The outcome indicators included ASES score, Constant score, VAS score, UCLA score, and re-tear rate.

For arthroscopic rotator cuff repair, double-row repair is significantly better than single-row repair in terms of ASES score, UCLA, and risk of re-tear. This may be because, compared with single-row repair, double-row repair increases the contact area between tendon and bone, increases the coverage area of ​​rotator cuff "footprints", and improves the maximum load and fixation strength at the suture site, contributing to better restoring the rotator cuff structure. Compared with the other methods, single-row repair can get better Constant scores. The performance of suture bridge repair is not ideal in the five outcome indicators. Previous Meta-analysis [61] showed that suture bridge repair shows better biomechanical properties than single-row repair and obtains better footprint coverage in in vitro studies. However, suture bridge repair also makes the tension on the inner row too concentrated, weakens the connection at the tendon junction, and increases the risk of muscle atrophy and postoperative re-tear.

Meanwhile, the present study also found that PRP therapy had better performance in ASES score, VAS score, and risk of re-tea. Moreover, in terms of UCLA and Constant scores, there was no significant statistical difference between PRP therapy and the other interventions. This finding confirms the effectiveness of PRP therapy for rotator cuff injuries. To date, numerous studies have analyzed the effects of PRP in enhancing rotator cuff repair. A study by Li et al. [42] found that PRP therapy resulted in lower re-tear rates during 2-year follow-up after rotator cuff injury. Contrary to our conclusions, this study found no difference in UCLA scores at 3, 6, and 24 months after surgery. Another Meta-analysis regarding the use of PRP reviewed seven randomized controlled trials published between 2013 and 2018 and found that patients with PRP therapy had significantly lower re-tear rates and improved UCLA scores compared to those with no use of PRP [62]. Therefore, although PRP can improve the postoperative UCLA scores, there is no significant difference compared with the other surgical methods. However, it should be noted that in a relevant study, PRP has been divided into four categories: pure PRP, leukocyte and PRP, pure platelet-rich fibrin, and leukocyte-PRP and platelet-rich fibrin [63]. Different kinds of PRP may lead to inconsistent results.

As for bone marrow stimulation techniques, a recent meta-analysis [63] showed that bone marrow stimulation could reduce postoperative recurrence rates but not significantly improve functional outcomes compared with traditional repair methods. However, the present study found that bone marrow stimulation achieved the best Constant score in postoperative prognosis. And in terms of VAS score and risk of re-tear, after eliminating inconsistency, the bone marrow stimulation technique was also ranked top. Since mesenchymal stem cells generated in the foramen after microfracture may promote better histological healing of the repaired tendon [64], Beitzel conducted a retrospective analysis of the use of bone marrow mesenchymal stem cells to repair rotator cuff injuries. In the seven studies included, bone marrow mesenchymal stem cells were shown to promote healing but had no marked efficacy in one study. This is consistent with the results of our study [65, 66]. In addition, some basic and clinical studies have shown that PRP can stimulate local osteogenesis and promote the proliferation of mesenchymal stem cells, which is effective in the treatment of delayed healing or nonunion [67]. Chong found that at the early stage of tendon injury, injection of bone marrow mesenchymal stem cells could effectively promote tendon healing and improve its biomechanical properties.

The appropriate treatment regimen with effective rehabilitation can make patients achieve better outcomes [68]. Before implementing a rehabilitation regimen after rotator cuff repair, precautions should be given to the selection of surgical method, patient’s biological parameters, patient’s expectations for postoperative work, exercise, daily activities or recovery, and bone-to-tendon or tendon-to-tendon biological healing time. And based on these indicators, a personalized rehabilitation plan will be specified.

Based on the results of these five clinical outcome indicators, we concluded that PRP therapy is the most effective method for rotator cuff repair and double-row repair and bone marrow stimulation also have better prognostic outcomes. However, the overall therapeutic effect of single-row or suture bridge repair is relatively poor and both of them are not recommended for rotator cuff repair.

## Data Availability

All the data and materials were included within the manuscript.
